# Pediatric Intensive Care Unit Admissions for COVID-19: Insights Using State-Level Data

**DOI:** 10.1155/2020/9680905

**Published:** 2020-11-18

**Authors:** Rohit S. Loomba, Enrique G. Villarreal, Juan S. Farias, Ronald A. Bronicki, Saul Flores

**Affiliations:** ^1^Division of Pediatric Cardiac Critical Care, Advocate Children's Hospital, Chicago, IL, USA; ^2^Department of Pediatrics, Chicago Medical School/Rosalind Franklin School of Medicine and Science, Chicago, IL, USA; ^3^Tecnologico de Monterrey, Escuela de Medicina y Ciencias de la Salud, Monterrey, Nuevo Leon, Mexico; ^4^Sections of Critical Care Medicine and Cardiology, Texas Children's Hospital, Houston, TX, USA; ^5^Department of Pediatrics, Baylor College of Medicine, Houston, TX, USA

## Abstract

**Introduction:**

Intensive care has played a pivotal role during the COVID-19 pandemic as many patients developed severe pulmonary complications. The availability of information in pediatric intensive care units (PICUs) remains limited. The purpose of this study is to characterize COVID-19 positive admissions (CPAs) in the United States and to determine factors that may impact those admissions.

**Materials and Methods:**

This is a retrospective cohort study using data from the COVID-19 Virtual Pediatric System (VPS) dashboard containing information regarding respiratory support and comorbidities for all CPAs between March and April 2020. The state-level data contained 13 different factors from population density, comorbid conditions, and social distancing score. The absolute CPA count was converted to frequency using the state's population. Univariate and multivariate regression analyses were performed to assess the association between CPA frequency and admission endpoints.

**Results:**

A total of 205 CPAs were reported by 167 PICUs across 48 states. The estimated CPA frequency was 2.8 per million children in a one-month period. A total of 3,235 tests were conducted of which 6.3% were positive. Children above 11 years of age comprised 69.7% of the total cohort and 35.1% had moderated or severe comorbidities. The median duration of a CPA was 4.9 days (1.25–12.00 days). Out of the 1,132 total CPA days, 592 (52.2%) involved mechanical ventilation. The inpatient mortalities were 3 (1.4%). Multivariate analyses demonstrated an association between CPAs with greater population density (beta coefficient 0.01, *p* < 0.01). Multivariate analyses also demonstrated an association between pediatric type 1 diabetes mellitus with increased CPA duration requiring advanced respiratory support (beta coefficient 5.1, *p* < 0.01) and intubation (beta coefficient 4.6, *p* < 0.01).

**Conclusions:**

Inpatient mortality during PICU CPAs is relatively low at 1.4%. CPA frequency seems to be impacted by population density. Type 1 DM appears to be associated with increased duration of HFNC and intubation. These factors should be included in future studies using patient-level data.

## 1. Introduction

During the pandemic, the world has been subdued by the rapid worldwide spread of the novel Severe Acute Respiratory Syndrome Coronavirus (SARS-CoV-2). On March 11, 2020, the World Health Organization declared the disease caused by the novel virus (COVID-19) a pandemic health emergency for the first time since the swine flu (H1N1) in 2009 [1]. As of October 2020, there are more than 38.3 million people infected, with more than 1,090,000 deaths worldwide [2]. The United States (US) is the most impacted nation with more than 8 million people infected and more than 220,000 deaths [3]. Hospitals in areas where the pandemic has caused devastation continue to struggle as many challenges remain unmet due to the speed of transmission, the lack of accurate knowledge regarding the benefits or pitfalls of the current available therapies, and the uncertainty of being able to provide adequate care if the rate of transmission continues.

The availability of intensive level of care has played a pivotal role, as many patients developed severe pulmonary complications. Shekerdemian and colleagues described the burden of COVID-19 infection in North America's PICUs. This early study describes that severe illness is less frequent than in adults and that prehospital comorbidities are important factors of severity [4]. González-Dambrauskas et al. described a preliminary report of the CAKE (Critical Coronavirus and Kids Epidemiologic) study that involves 60 centers in 20 countries from Europe and the Americas. This international report provided clinical and laboratory data of 17 patients admitted to the PICU and concluded that COVID-19 in children is a variable disease but with general better outcomes than adults [5]. However, there is limited information on COVID-19 positive admissions (CPAs) to pediatric intensive care units (PICUs) regarding patient characteristics, respiratory support required, and their impact on outcomes. Therefore, the purpose of this study is to better characterize CPAs to PICUs in the US and to determine factors that may impact those admissions.

## 2. Methods

This study utilized only publicly available, deidentified, state-level data. As such, no institutional review board review or approval was sought.

### 2.1. Endpoint Identification and Data Collection

The following data was identified for collection regarding the CPAs themselves: number, duration, need for various ventilatory support measures, severity of comorbidities, and the total number of COVID-19 tests conducted. The following data was collected regarding US states: pediatric population, state population (pediatric and adult) density, air and drinking water quality, average temperature, average ultraviolet index, prevalence of pediatric obesity, type 1 diabetes mellitus (DM) and asthma, the proportion of children who smoke cigarettes, received the influenza vaccine, had health insurance, received home health care, race, percent of households with children below the poverty line, highest education level of adults in homes with children, and the social distancing score by global positional satellite data (Supplementary Table 1).

The data regarding the CPAs themselves was collected from the publicly available COVID-19 dashboard provided by the Virtual Pediatric System (VPS), which collects data from 167 out of 5,100 PICUs in most states of the US (all states except Maine and Wyoming). COVID-19 data was collected from March 14 through April 14, 2020, in order to represent one full month of data [6]. Data regarding number of centers, number of tests, and number of CPAs was captured in absolute counts. Data regarding the duration of CPA was collected in days. The respiratory support modalities for which data was available were room air (RA) and nasal cannula (NC), and for the advanced respiratory support modalities (i.e., other than RA and NC), there was available data for high-flow nasal cannula (HFNC), noninvasive positive pressure ventilation (NIPPV), mechanical conventional ventilation (MCV), high-frequency oscillatory ventilation (HFOV), and extracorporeal membrane oxygenation (ECMO) and was captured in duration (days) of their use. Data regarding severity of comorbidities is reported in the VPS dashboard, and the percentage of CPAs with moderate or severe degree of comorbidities was collected.

State-wide data for the analyses were collected from a variety of sources with the complete list of sources provided as Supplementary Material 1. Children's population data and pediatric comorbidity data were obtained from 2018, as these were the most recent and comprehensive data available. The sources for these other data points were generally US government-based efforts to capture state-level data on various medical issues; however, not all states reported data for all the endpoints (Supplementary Table 2).

Endpoints were assigned to the authors for collection. One author was responsible for collecting data for each state for the variables assigned. Once these data were collected, a different author, who did not primarily collect data for that specific endpoint, verified the numbers for accuracy. Finally, values in the top and bottom 10^th^ percentile were identified and verified by a third author.

### 2.2. Statistical Analyses

As the data was collected for each state and intended for state-level analyses and each state has a different pediatric population, the absolute numbers of CPAs for each state were not directly comparable. Thus, the absolute CPA count for each state was first converted to a frequency of CPAs per 1,000,000 children using the specific state's population. This CPA frequency was then used as the dependent variable in a series of single-independent variable linear regressions to determine the univariate association between CPA frequency and the other predictors. Multivariate regression was conducted with CPA frequency as the dependent variable and with other variables entered as independent variables. Forward stepwise regression was utilized with the model with the greatest *R*-squared value being used for the analyses.

Next, a composite endpoint called “percent of PICU days requiring advanced respiratory support” was created. This consisted of the total duration of HFNC, NIPPV, MCV, HFOV, and ECMO divided by the total PICU admission duration. This was then modeled similarly to CPA frequency. Next, a composite outcome called “percent of PICU days requiring intubation” was created. This consisted of the total duration of MCV and HFOV divided by the total PICU admission duration. This, too, was then modeled similarly as CPA frequency. Lastly, an endpoint called “PICU duration per admission” was created for each state and consisted of the total CPA PICU duration for that specific state divided by the number of CPAs reported by that state. This was also then modeled similarly to CPA frequency.

All statistical analyses were done using the user-coded, syntax-based interface of SPSS Version 23.0. All statistical analyses were done with state-level data. A *p* value of 0.05 was considered statistically significant, and any use of the word significant hereon in the manuscript refers to “statistically significant.”

## 3. Results

### 3.1. COVID-19 Pediatric Intensive Care Unit Admission Characteristics

**Figure 1:**
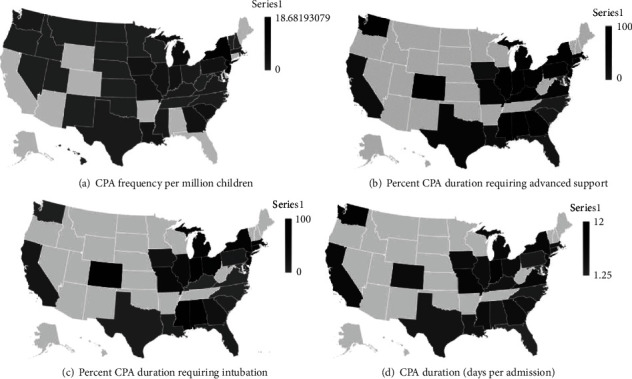
COVID-19 pediatric intensive care unit admission characteristics by state. (a) COVID-19 pediatric admission frequency per million children. (b) Percent of COVID-19 pediatric admission duration requiring advanced respiratory support. (c) Percent of COVID-19 pediatric admission requiring intubation. (d) COVID-19 pediatric admission duration: days per admission. CPA indicates COVID-19 pediatric admission.

A total of 205 CPAs were reported by 167 PICUs across 48 states. The states not represented in the VPS dataset were Maine and Wyoming. By using an estimated pediatric population of the US of 72,886,669, the frequency of CPAs was 2.8 per million children (Figure 1(a)). The median CPA frequency for a state was 0.66 with a range of 0 to 18.68. A total of 3,235 tests were conducted by all reporting sites, resulting in 6.3% of total tests resulting positive (Table 1). The total CPA duration for all 205 CPAs was 1,132 days. Out of the 1,132 CPA days, 309 days (27.3%) were on nasal cannula or room air, 129 (11.6%) consisted of HFNC, 83 (7.3%) of NIPPV, 592 (52.2%) of MCV, 6 of HFOV, and 13 (1.6%) of ECMO (Figures 1(b) and 1(c)). The median duration of a CPA was 4.9 days, with a range of 1.25 to 12.00 days (Figure 1(d)). Of the 205 CPAs, 3 (1.4%) ended in inpatient mortality.

### 3.2. COVID-19 Pediatric Intensive Care Unit Admission Frequency, Univariate and Multivariate Analyses

Univariate analyses with CPA frequency as the dependent variable demonstrated the following factors to be significantly associated with increasing CPA frequency: greater population density (beta coefficient 0.01, *p* < 0.01) and increased percent of children receiving the influenza vaccination (beta coefficient 0.17, *p* = 0.01). None of the other state predictor variables were associated with significant reductions in CPA frequency (Table 2). Multivariate regression analysis only demonstrated the following to be associated with increased CPA frequency: population density (beta coefficient 0.1, *p* < 0.01). The *R*-squared value was 0.28 (Table 3).

### 3.3. COVID-19 Pediatric Intensive Care Unit Admission Requirement of Advanced Respiratory Support, Univariate and Multivariate Analyses

Univariate analyses with the percentage of CPA duration requiring advanced respiratory support as the dependent variable demonstrated the following factor to be associated with a significant increase in the duration: increased prevalence of type 1 DM (beta coefficient 4.52, *p* = 0.01). The following was associated with a decrease in this duration: ultraviolet light index (beta coefficient -12.00, *p* = 0.04) (Table 2). Multivariate regression analysis demonstrated the following to be associated with increased percentage of CPA duration requiring advanced respiratory support: higher prevalence of type 1 diabetes (beta coefficient 5.1, *p* < 0.01) and higher precipitation (beta coefficient 8.3, *p* < 0.01). The *R*-squared value was 0.83 (Table 3).

### 3.4. COVID-19 Pediatric Intensive Care Unit Admission Requirement of Intubation, Univariate and Multivariate Analyses

Univariate analyses with the percent of CPA duration requiring intubation as the dependent variable demonstrated the following factor to be associated with a significant increase in the duration: increased prevalence of type 1 DM (beta coefficient 4.89, *p* < 0.01). There were no factors associated with a significant decrease in percentage of CPA duration requiring intubation (Table 2). Multivariate regression analysis demonstrated the following to be associated with increased percentage of CPA duration requiring intubation: prevalence of type 1 diabetes (beta coefficient 4.6, *p* < 0.01). The *R*-squared value was 0.40 (Table 3).

### 3.5. COVID-19 Pediatric Intensive Care Unit Admission Duration, Univariate and Multivariate Analyses

Univariate analyses with CPA duration as the dependent variable demonstrated the following factors to be significantly associated with decreased admission duration: increased ultraviolet light index (beta coefficient -0.78, *p* = 0.04) and increased average temperature (-0.09, *p* = 0.04). There were no factors associated with a significant increase in CPA duration (Table 2). None of the variables remained statistically significant in multivariate analysis for CPA duration. Thus, no model is presented.

### 3.6. Power Analyses

Multivariate regression analysis for CPA frequency and percentage of CPA duration requiring intubation were not adequately powered. Multivariate regression analysis for percentage of CPA duration requiring advanced respiratory support was adequately powered. Effect sizes and number of subjects to achieve power for the multivariate analyses are described in Supplementary Table 3.

## 4. Discussion

This study identified factors that impact the frequency of CPAs to the PICU in the United States. The main finding of this study is that inpatient mortality during PICU CPAs is relatively low at 1.4%, CPA frequency appears to be impacted by population density, and type 1 DM appears to be associated with increased duration of HFNC and intubation with slightly over half of CPAs required intubation. In addition, the burden of COVID-19 in children has not been nearly as substantial as in the adults, with a CPA frequency of 2.8 per million children. The highest 5-day rolling census average noted in the VPS dashboard was 90 CPAs with a median CPA duration over the study period of 4.9 days similar to a recent publication [4]. If this number is doubled to account for PICUs not reporting to VPS, that would imply a maximum CPA burden of 180 in a single day. Considering that there are approximately 5,100 PICU beds in the US and a down trending daily PICU census and new CPA numbers reported in the VPS dashboard, it is unlikely to become a burden on the system during the study time. This is an important finding as a recent model reported a possibility of a higher burden of disease without providing a time frame for cases to present [7].

These findings are consistent with current national and international trends. Early in April 2020, the Morbidity and Mortality Weekly Report stated that pediatric cases represent 1.7% of the total cases in the US [8], whereas in Italy and China, the cases represented 1.2% and 2%, respectively [9, 10]. Furthermore, the majority of pediatric patients presented with mild symptoms having a complete recovery within 1 to 2 weeks after the onset of illness [11]. A study by Dong and colleagues reported in a cohort of 2,135 confirmed and suspected pediatric COVID-19 cases that 5.8% were severe or critical and there was 1 mortality [12]. As of October 15, 2020, the “Children and COVID-19: State-Level Data Report” by the American Academy of Pediatrics reported 741,891 total cumulative pediatric COVID-19 cases in the US (representing 10.9% of the total cases in the US). Children were 1.7% of total hospitalizations (5,353 children hospitalized out of 314,715 total hospitalizations in the US), and between 0.5 and 7.2% of all pediatric COVID-19 cases resulted in hospitalization [13].

The association with state population density makes intuitive sense as urban centers potentiate a rapid spread of an infectious disease, as well as a greater number of global travel routes [14]. Though it was reported that influenza immunization rates are higher in urban areas [15], the role of influenza immunization in the COVID-19 transmission requires further investigation as recent studies have described the phenomenon of virus interference in which an immunization against one virus may increase the risk of illness from other viruses [16]. A recent study by Wolff and colleagues found in a cohort of 6,000 patients that influenza immunization increased the risk of illness from nonpandemic coronavirus (odds ratio of 1.36; 95% confidence interval 1.14 to 1.63, *p* < 0.01) [17]. Similarly, Rikin and colleagues identified an increased risk of noninfluenza respiratory illness in children who received the influenza vaccine when compared to those who did not (hazard ratio 1.65, 95% confidence interval 1.14 to 2.38). Future studies on pediatric COVID-19 should include influenza vaccination as one of the variables of interest.

There is limited data on the association between environmental temperature or ultraviolet index and the burden of COVID-19 in children. Our findings showed an association between an increase in ultraviolet index and temperature and a reduction in duration of advanced respiratory support (i.e., increased from room air or nasal cannula). An association was identified by Gunthe and colleagues with lower environmental temperatures between 5 and 15 degree Celsius (41 to 59 degree Fahrenheit) and a low ultraviolet index of 2.5 with an increased number of COVID-19 cases [18]. These findings are in alignment with prior studies on SARS-CoV-1 where high temperature and high humidity decreased its survival on contaminated surfaces [19]. Further studies are warranted to determine the implications of environmental factors in SARS-CoV-2 life cycle.

Our study findings showed an association between PICU CPAs requiring intubations and prevalence of type 1 DM. A study by Shekerdemian and colleagues identified in a cohort of 48 confirmed COVID-19 critically ill pediatric cases that 4 (8%) are with type 1 DM with 3 of them presenting with diabetic ketoacidosis [4]. Future studies should focus on determining the role of DM in the pathogenesis and severity of COVID-19 in children.

As the mechanisms of viral spread remain largely undefined, countries have engaged in the implementation of social distancing as the main preventive measure. However, the role of children in spreading SARS-CoV-2 is highly speculated and presents conflicting answers with some reports stating similar virus spreading between children and adults [20], whereas other reports from Wuhan and Shanghai state that children are less likely than adults to spread the virus [21]. Some European nations like Sweden have faced the current pandemic with alternate strategies including more lenient social distancing efforts and have not had, to date, severely adverse outcomes as a result in children [22]. Though the results of our analysis may support the latter, since children represent a small fraction of confirmed COVID-19 cases, further investigation is necessary to determine the impact of asymptomatic carriage and low identification rate in children. There is limited information to determine the impact of social distancing and lockdowns during this pandemic; previously published modeling data demonstrates that, in certain circumstances, social distancing may have no impact on the illness and in certain circumstances may have negative population outcomes [23]. Studies of transmission in schools will be important as schools get ready to reopen. Meanwhile, the population should follow the recommendations from local municipalities.

These analyses offer an early look at COVID-19 PICU admissions and offer some descriptive insight into these admissions as well as use state-wide data to investigate associations between CPA characteristics with comorbidities, environmental factors, and socioeconomic factors. While these analyses offer novel data regarding CPAs in the US, they are not without their limitations. Firstly, all the data is state-wide data as no patient-level data was used. Thus, the 48 states with reported data are the cases, not the 205 individual CPAs. The data regarding comorbidities, environmental factors, and socioeconomic factors is also state-level data and is not specific to the CPAs. Rather, the information used is publicly available data about the children or families in the respective state. Additionally, the multivariate analyses are underpowered, and thus, the univariate analyses in this study offer the more meaningful data. It must be kept in mind that all significant findings in this study are simply associations as causation cannot be inferred due to the study design. Thus, findings such as those related to immunizations and social distancing cannot be considered to be causal and should not be utilized to impact decision-making.

Despite the limitations outlined, these analyses offer helpful information that may be used to assist during the consideration as to what factors need more clarification from future studies with patient-level data. These results also offer a cross-sectional view regarding US PICU CPAs not previously reported. The use of state-level data allowed for analyses relatively early, before aggregate, multicenter, patient-level data-based studies can be completed; thus, it can be used while the pandemic is still occurring. Furthermore, these analyses also offer insight into some of the factors that should be included in future studies and may also be helpful for resource allocation planning and CPAs.

## 5. Conclusion

Inpatient mortality during PICU CPAs is relatively low at 1.4%. CPA frequency seems to be impacted by population density. Type 1 DM appears to be associated with increased duration of HFNC and intubation. These factors should be included in future studies using patient-level data.

## Figures and Tables

**Table 1:** Demographic characteristics and outcomes of children treated in pediatric intensive care units for COVID-19 in the United States of America.

Characteristics	Values representing state-level data^a^
Age	
Under 2 years of age	31 (15.1%)
Between 2 and 11 years of age	32 (15.6%)
Between 11 and 18 years of age	142 (69.7%)
Comorbidities	
Mild comorbidities	70 (33.6%)
Moderate or severe comorbidities	73 (35.1%)
Number of site reporting	2.0 (1.0 to 23.0)
Number tested	31.0 (0 to 575.0)
CPA frequency per 1,000,000	0.8 (0 to 18.6)
Duration of HFNC (days)	1.0 (0 to 58.0)
Duration of NIPPV (days)	0 (0 to 38.0)
Duration of MCV (days)	4.5 (0 to 308.0)
Duration of HFOV (days)	0 (0 to 5.0)
Duration of ECMO (days)	0 (0 to 7.0)
CPA days total^b^	15.5 (2.0 to 498.0)
CPA days per admission^b^	4.9 (1.2 to 12.0)

Abbreviations: CPA: COVID-19 positive admission; HFNC: high-flow nasal cannula; NIPPV: noninvasive positive pressure ventilation; MCV: mechanical conventional ventilation; HFOV: high-frequency oscillatory ventilation; ECMO: extracorporeal membrane oxygenation. ^a^Data are presented as median (IQR) or number of patients (percentage, %), as state-level data in the United States of America. ^b^CPA days total refers to the sum of admission days by state. CPA days per admission refers to the actual days by each patient admitted by state.

**Table 2:** Univariate analysis: association between pediatric population characteristics and COVID-19 positive admissions.

	CPA frequency		Percent CPA duration requiring HFNC or more		Percent CPA duration requiring intubation (MCV or HFOV)		Number of PICU days per CPA	
	Beta coefficient	*p* value^a^	Beta coefficient	*p* value^a^	Beta coefficient	*p* value^a^	Beta coefficient	*p* value^a^
Population density	0.008	0.004	-0.011	0.685	0.011	0.647	-0.00008	0.996
Urban air quality	0.032	0.404	-0.209	0.305	0.098	0.856	0.07	0.864
Drinking water quality	-0.055	0.147	-0.207	0.765	-0.309	0.636	0.032	0.48
UV index	-0.614	0.182	-12.008	0.04	-5.821	0.308	-0.782	0.044
Average precipitation	0.421	0.185	8.94	0.084	6.308	0.203	0.116	0.743
Average temperature	0.018	0.708	-1.296	0.064	-0.768	0.256	-0.093	0.045
Percent households below poverty line	0.053	0.612	-0.399	0.784	0.348	0.801	-0.083	0.39
Percent households with below high school as the highest adult education level	0.111	0.502	-3.926	0.166	-3.182	0.238	-0.032	0.866
Percent pediatric obesity	0.067	0.614	-2.187	0.28	-0.391	0.84	-0.179	0.179
Percent pediatric type 1 DM	-0.052	0.68	4.525	0.012	4.899	0.003	0.116	0.356
Percent pediatric asthma	0.175	0.779	-3.707	0.564	1.534	0.821	-0.079	0.851
Percent high schoolers currently smoking	-1.48	0.524	-0.726	0.836	-3.397	0.3	-0.269	0.242
Percent pediatric flu shot received	0.177	0.019	0.252	0.814	-0.22	0.828	0.026	0.72
Percent pediatric who have health insurance	0.24	0.294	2.368	0.438	2.604	0.366	0.225	0.264
Percent pediatric who have a medical home	-0.098	0.449	1.486	0.425	0.715	0.687	0.066	0.595
Percent Hispanic	0.015	0.725	-0.37	0.535	-0.468	0.405	0.001	0.99
Percent white	-0.038	0.256	0.799	0.149	0.587	0.266	0.02	0.589
Percent black	0.093	0.072	-0.741	0.279	0.113	0.863	-0.05	0.27
Percent other race	-0.027	0.638	0.376	0.848	-2.903	0.109	0.156	0.224
Social distancing score	0.178	0.848	17.57	0.196	3.558	0.786	1.664	0.061
Moderate or severe comorbidity^b^			0.088	0.686	-0.009	0.965	0.014	0.33

Abbreviations: CPA: COVID-19 positive admission; HFNC: high-flow nasal cannula; MCV: mechanical conventional ventilation; HFOV: high-frequency oscillatory ventilation; PICU: pediatric intensive care unit; UV: ultraviolet; DM: diabetes mellitus. ^a^*p* value < 0.05 was considered statistically significant. ^b^No data available to assess moderate or severe comorbidity with admission frequency.

**Table 3:** Multivariate regression analyses.

*Increased CPA frequency*				
Covariates included in analysis	Beta coefficient	*p* value^a^	*R*-squared	Collinearity
Population density	0.1	<0.01	0.28	No
*Increased percent CPA duration requiring HFNC or more*				
Covariates included in analysis	Beta coefficient	*p* value^a^	*R*-squared	Collinearity
Percent pediatric type 1 DM	5.1	<0.01	0.83	No
Higher precipitation	8.3	<0.01	0.83	No
*Increased CPA duration requiring intubation (MCV or HFOV)*				
Covariates included in analysis	Beta coefficient	*p* value^a^	*R*-squared	Collinearity
Percent pediatric type 1 DM	4.6	<0.01	0.40	No

Abbreviations: CPA: COVID-19 positive admission; HFNC: high-flow nasal cannula; DM: diabetes mellitus; MCV: mechanical conventional ventilation; HFOV: high-frequency oscillatory ventilation. ^a^*p* value < 0.05 was considered statistically significant.

## Data Availability

Deidentified individual participant data will not be made available.

## Supplementary Materials

Supplementary materials Supplementary Table 1 Information resource for state-wide data collection. Supplementary Table 2 States that reported data for each endpoint. Supplementary Table 3 Power analyses for multivariate regression analysis.
