# Supplementary material for: Pediatric Intensive Care Unit Admissions for COVID-19: Insights Using State-Level Data
Source: Int J Pediatr. 2020 Nov 18;2020:9680905. doi: 10.1155/2020/9680905 (PMC7704189; doi:10.1155/2020/9680905)
Supplement: Supplementary materials — Supplementary Table 1 Information resource for state-wide data collection. Supplementary Table 2 States that reported data for each endpoint. Supplementary Table 3 Power analyses for multivariate regression analysis. [file 9680905.f1.zip › Supplementary Table 1.docx]

**Supplementary Table 1. Information Resource for state-wide data collection**

| **Outcome** | **Information Resource** |
| --- | --- |
| 1. PICU admissions | https://myvps.org |
| 2. Pediatric population (to create point estimate) | https://datacenter.kidscount.org/data/map/99-total-population-by-child-and-adult-populations?loc=1&loct=1#2/any/false/false/37/39/416/Orange/-10669123,4079423.5,2,159.2727279663086,205.09091186523438 |
| 3. Population density (to see if more dense places have higher rates of critical illness) | https://worldpopulationreview.com/states/state-densities/ |
| 3. Population density (to see if more dense places have higher rates of critical illness) | https://worldpopulationreview.com/states/state-densities/ |
| 4. Urban air quality | https://www.usnews.com/news/best-states/rankings/natural-environment/air-water-quality |
| 5. Drinking water quality (this is a score for each state and see if this makes a difference) | https://www.usnews.com/news/best-states/rankings/natural-environment/air-water-quality |
| 6. UV index (collect average for march from the graph. can estimate) | https://www.epa.gov/sunsafety/sun-safety-monthly-average-uv-index |
| 7. Precipitation | https://www.ncdc.noaa.gov/cag/statewide/rankings/50/pcp/202003 |
| 8. Temperature (capture march in Fahrenheit) | <https://www.ncdc.noaa.gov/cag/statewide/rankings/50/tavg/202003> |
| 9. Percent of pediatric households below 100% poverty line | https://www.childhealthdata.org/browse/survey/allstates?q=7265 |
| 10. Highest education level of adults | https://www.childhealthdata.org/browse/survey/allstates?q=7267 |
| 11. Pediatric obesity % | https://www.childhealthdata.org/browse/survey/allstates?q=7297 |
| 12. Pediatric asthma % | https://www.cdc.gov/asthma/brfss/2018/child/tableC1.html |
| 13. Pediatric diabetes % | https://www.cdc.gov/pcd/issues/2018/18_0323.htm |
| 14. Pediatric current smoker % | https://www.cdc.gov/tobacco/about/osh/state-fact-sheets/index.htm |
| 15. Pediatric flu vaccination % | https://www.cdc.gov/flu/fluvaxview/coverage-1718estimates-children.htm |
| 16. Pediatric health insurance | https://www.childhealthdata.org/browse/survey/allstates?q=6943 |
| 17. Pediatric medical home | https://www.childhealthdata.org/browse/survey/results?q=7097&r=1 |
| 18. Race | https://www.childhealthdata.org/browse/survey/allstates?q=7259 |
| 19. Social distancing score by GPS | https://www.unacast.com/covid19/social-distancing-scoreboard |

*Abbreviations: PICU, Pediatric Intensive Care Unit; UV, Ultraviolet; GPS, Global Positioning System.*
