# Supplementary material for: Pediatric Intensive Care Unit Admissions for COVID-19: Insights Using State-Level Data
Source: Int J Pediatr. 2020 Nov 18;2020:9680905. doi: 10.1155/2020/9680905 (PMC7704189; doi:10.1155/2020/9680905)
Supplement: Supplementary materials — Supplementary Table 1 Information resource for state-wide data collection. Supplementary Table 2 States that reported data for each endpoint. Supplementary Table 3 Power analyses for multivariate regression analysis. [file 9680905.f1.zip › Supplementary Table 2.docx]

**Supplementary Table 2. States that reported data for each outcome**

| **Outcomes** | **States that reported data** |
| --- | --- |
| PICU Admissions | All states, except Maine^a^ and Wyoming^b^. |
| PICU Admission Mortality | All states, except Alaska, Arizona, Arkansas, Hawaii, Idaho, Kansas, Maine^a^, Minnesota, Montana, Nebraska, Nevada, New Hampshire, New Mexico, North Dakota, Oklahoma, Oregon, Rhode Island, South Dakota, Tennessee, Utah, Vermont, West Virginia, Wisconsin and Wyoming^b^. |
| PICU Days | All states, except Alaska, Arizona, Arkansas, Hawaii, Idaho, Kansas, Maine^a^, Minnesota, Montana, Nebraska, Nevada, New Hampshire, New Mexico, North Dakota, Oklahoma, Oregon, Rhode Island, South Dakota, Tennessee, Utah, Vermont, West Virginia, Wisconsin and Wyoming^b^. |
| ECMO Days | All states, except Alaska, Arizona, Arkansas, Hawaii, Idaho, Kansas, Maine^a^, Minnesota, Montana, Nebraska, Nevada, New Hampshire, New Mexico, North Dakota, Oklahoma, Oregon, Rhode Island, South Dakota, Tennessee, Utah, Vermont, West Virginia, Wisconsin and Wyoming^b^. |
| HFOV Days | All states, except Alaska, Arizona, Arkansas, Hawaii, Idaho, Kansas, Maine^a^, Minnesota, Montana, Nebraska, Nevada, New Hampshire, New Mexico, North Dakota, Oklahoma, Oregon, Rhode Island, South Dakota, Tennessee, Utah, Vermont, West Virginia, Wisconsin and Wyoming^b^. |
| Conventional Ventilator | All states, except Alaska, Arizona, Arkansas, Hawaii, Idaho, Kansas, Maine^a^, Minnesota, Montana, Nebraska, Nevada, New Hampshire, New Mexico, North Dakota, Oklahoma, Oregon, Rhode Island, South Dakota, Tennessee, Utah, Vermont, West Virginia, Wisconsin and Wyoming^b^. |
| NIV^3^ Days | All states, except Alaska, Arizona, Arkansas, Hawaii, Idaho, Kansas, Maine^a^, Minnesota, Montana, Nebraska, Nevada, New Hampshire, New Mexico, North Dakota, Oklahoma, Oregon, Rhode Island, South Dakota, Tennessee, Utah, Vermont, West Virginia, Wisconsin and Wyoming^b^. |
| HFNC^4^ Days | All states, except Alaska, Arizona, Arkansas, Hawaii, Idaho, Kansas, Maine^a^, Minnesota, Montana, Nebraska, Nevada, New Hampshire, New Mexico, North Dakota, Oklahoma, Oregon, Rhode Island, South Dakota, Tennessee, Utah, Vermont, West Virginia, Wisconsin and Wyoming^b^. |
| Population density | All states, except Maine^a^ and Wyoming^b^. |
| Urban air quality | All states, except Maine^a^ and Wyoming^b^. |
| Drinking water quality | All states, except Maine^a^ and Wyoming^b^. |
| UV index | All states, except Alaska, Maine^a^ and Wyoming^b^. |
| Average precipitation | All states, except Hawaii, Maine^a^ and Wyoming^b^. |
| Average temperature | All states, except Hawaii, Maine^a^ and Wyoming^b^. |
| Percent households below poverty line | All states, except Maine^a^ and Wyoming^b^. |
| Percent households below high school as highest level of education for the adults | All states, except Maine^a^ and Wyoming^b^. |
| Percent pediatric obesity | All states, except Maine^a^ and Wyoming^b^. |
| Percent pediatric type 1 diabetes | All states, except Maine^a^ and Wyoming^b^. |
| Percent pediatric asthma | All states, except Alabama, Alaska, Arizona, Arkansas, Colorado, Delaware, Idaho, Iowa, Louisiana, Maine^a^, North Carolina, North Dakota, Oklahoma, South Carolina, South Dakota, Tennessee, Texas, Virginia, Washington, West Virginia and Wyoming^b^. |
| Percent among high school youth current smoker | All states, except Maine^a^ and Wyoming^b^. |
| Percent pediatric flu vaccination received | All states, except Maine^a^ and Wyoming^b^. |
| Percent pediatric with health insurance | All states, except Maine^a^ and Wyoming^b^. |
| Percent by racial group | All states, except Maine^a^ and Wyoming^b^. |
| Social distancing score | All states, except Maine^a^ and Wyoming^b^. |

*Abbreviations: PICU, Pediatric Intensive Care Unit; ECMO, Extracorporeal Membrane Oxygenator; NIV, Noninvasive Ventilation; HFOV, High-Flow Nasal Cannula; UV: Ultraviolet.*

^a, b^ Maine and Wyoming where the only states that didn’t report data to the Virtual Pediatric Systems, LLC (VPS-LLC).
