# Supplementary material for: Pediatric Intensive Care Unit Admissions for COVID-19: Insights Using State-Level Data
Source: Int J Pediatr. 2020 Nov 18;2020:9680905. doi: 10.1155/2020/9680905 (PMC7704189; doi:10.1155/2020/9680905)
Supplement: Supplementary materials — Supplementary Table 1 Information resource for state-wide data collection. Supplementary Table 2 States that reported data for each endpoint. Supplementary Table 3 Power analyses for multivariate regression analysis. [file 9680905.f1.zip › Supplementary Table 3.docx]

**Supplementary Table 3. Power Analyses for Multivariate Regression Analysis**

| **Multivariate analysis** | **Effect Size** | **Power** | **Required number of subjects** | **Model adequately powered?** |
| --- | --- | --- | --- | --- |
| CPA frequency | Low effect size | 80% | 385 | No |
| Percentage of CPA duration requiring advanced respiratory support | Low effect size | 80% | 31 | Yes |
| Percentage of CPA duration requiring intubation | Median effect size | 80% | 49 | No |

*Abbreviations: CPA, COVID-19 Positive Admission*
